# Thai version of ACL return to sports after injury scale translated with cross-cultural adaptation provided the good validation in Thai patients who received ACL reconstruction

**DOI:** 10.1051/sicotj/2025009

**Published:** 2025-03-13

**Authors:** Teerapat Laddawong, Chaiyanun Vijittrakarnrung, Patarawan Woratanarat, Nadhaporn Saengpetch

**Affiliations:** 1 Department of Physical Therapy, Faculty of Allied Health Sciences, Thammasat University Pathumthani 12120 Thailand; 2 Department of Orthopedics, Faculty of Medicine Ramathibodi Hospital, Mahidol University Bangkok 10400 Thailand

**Keywords:** ACL-return to sports after injury scale, ACL reconstruction, Tempa scale of kinesiophobia, Cross-cultural adapted translation

## Abstract

*Purpose*: The Anterior Cruciate Ligament Return to Sports after Injury scale (ACL-RSI) has been translated and culturally adapted into the Thai version. This study aimed to evaluate the reliability and validity of the Thai ACL-RSI for athletes recovering from ACL reconstruction. *Methods*: This study was a cross-sectional study. Forward-backward translation, cultural adaptation, and validation of the Thai ACL-RSI were performed and tested in 40 athletes (8 females, 32 males; mean age 30.2 ± 7.32 years; mean body weight 70.7 ± 13.36 kg; mean height 170.1 ± 6.53 cm; mean body mass index 24.5 ± 3.74 kg/m^2^; mean time from surgery to evaluation 8.43 ± 1.83 months). Participants completed the translated Thai ACL-RSI and the validated Thai Tampa Scale of Kinesiophobia (TSK). The Thai ACL-RSI underwent content validity, internal consistency, reliability, and construct validity assessment. *Results*: The Thai ACL-RSI demonstrated commendable content validity (item-objective congruence index [IOC] 0.91), internal consistency (Cronbach’s alpha coefficient 0.84), and test-retest reliability (intraclass correlation coefficient [ICC] 0.75). There was a significant negative correlation with TSK (*r* = −0.67, *p* < 0.001). *Conclusion*: The Thai ACL-RSI is validated, reliable, and consistent with the Thai TSK. This instrument can potentially measure psychological factors influencing preparedness for sports participation after ACL reconstruction. The evaluation of return-to-sport readiness should involve a multidisciplinary approach, including surgeons, physiotherapists, and psychologists, to ensure a comprehensive assessment of physical, functional, and psychological factors.

## Introduction

Injuries to the anterior cruciate ligament (ACL) are a common cause of chronic knee instability. It causes a significant decline in performance and restricts participation in high-intensity physical activities, especially those involving pivoting and cutting movements [1]. ACL reconstruction (ACLR) has become a standard therapeutic intervention. The goal is the restoration of knee stability and the return to their pre-injury levels of performance [2]. Implementing an effective rehabilitation program is crucial not only for achieving the desired outcomes but also for maintaining patient motivation throughout the arduous process. Return to play (RTP) decision requires a comprehensive evaluation of multiple factors encompassing both physical and psychological dimensions [3, 4]. The criteria for a RTP decision include the accomplishment of reliable muscular strength, full range of motion, and the assessment of knee stability, as supported by Webster et al. [5]. Notably, the psychological aspect shows a significant relationship with overall recovery [6].

In 2008, Webster et al. developed the ACL Return to Sports after Injury (ACL-RSI) scale, which consists of 12 questions stratified into three domains: emotions (consisting of five items), self-efficacy (performance confidence, consisting of five items), and abilities (consisting of two risk assessments) [5]. This instrument has demonstrated high reliability and is commonly used to assess the psychological state of athletes prior to the resumption of training and competition. The original English version of the ACL-RSI has been translated into multiple languages, including Swedish, French, German, Dutch, and Turkish [7–10]. Muller et al. [11] discovered that the ACL-RSI scale possessed strong predictive abilities for the return to sport in patients who had undergone ACLR surgery for 6 months. This study hypothesized that translating the ACL-RSI into Thai would prevent potential misunderstandings and misinterpretations resulting from the use of an alternative validated Thai version of the kinesiophobia scoring system [12, 13]. The primary objective of this study was to translate the ACL-RSI from English to Thai and then validate its applicability within the Thai patient population. In addition, this study aimed to conduct a comprehensive evaluation of the Thai ACL-RSI’s numerous characteristics to determine the scale’s reliability and validity in the context of cross-cultural translation.

## Methods

**Figure 1:**
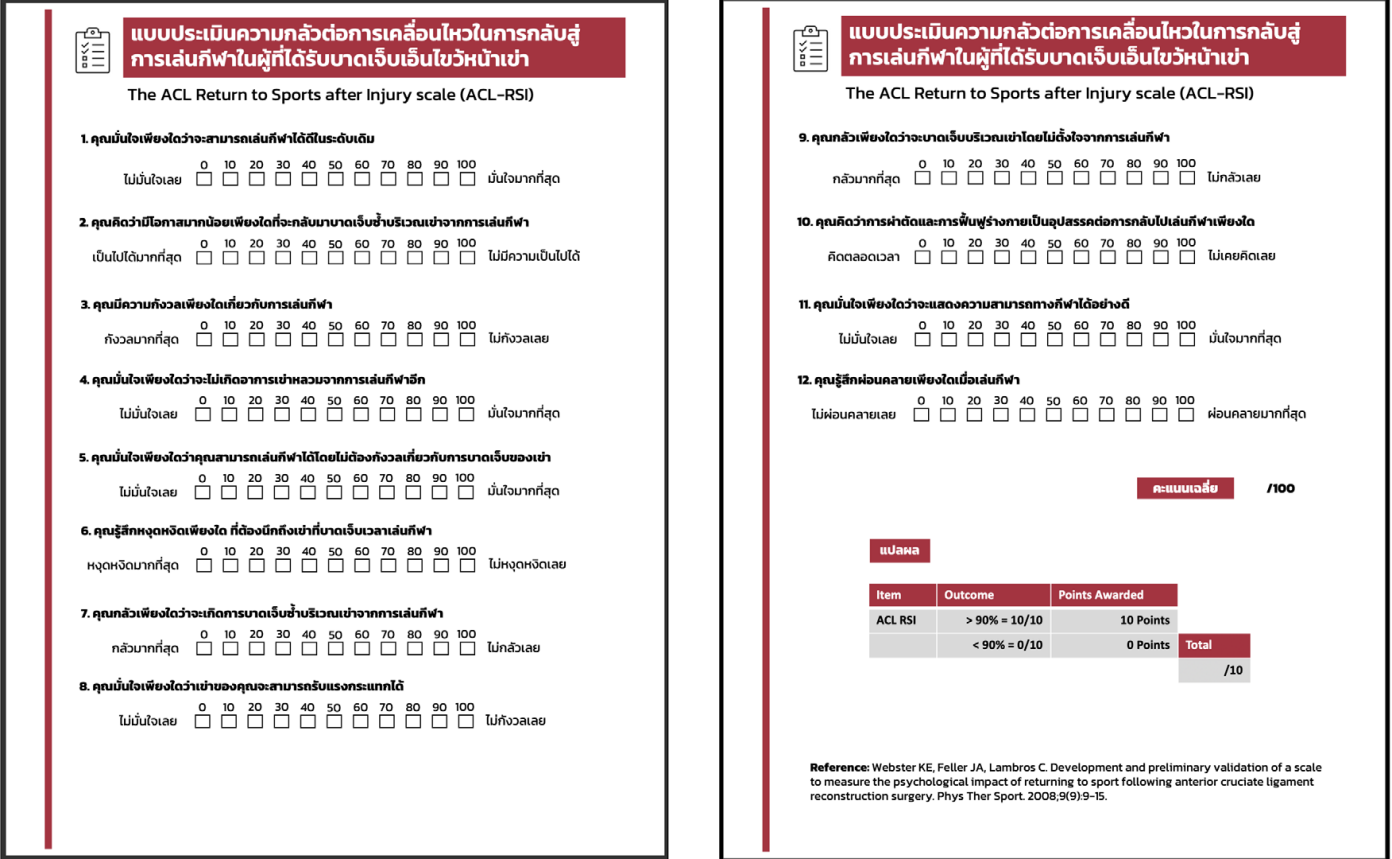
Final version of ACL-RSI scale in Thai.

The local ethics committee approved this cross-sectional study. A longitudinal, single-arm cohort was designed for this study with two phases [9]. Phase 1 involved translation and cross-cultural adaptation of the ACL-RSI, following Beaton’s and Harput’s methodology [9, 12]. Cross-cultural adaptation ensures that the translated version is not only linguistically accurate but also culturally relevant to Thai patients. This process minimizes potential misinterpretations due to cultural differences in language and expression. The translation process followed five structured steps:

- Step 1: Two independent Thai translators, one specialized in sports psychology and the other in English instruction, performed both literal and conceptual translations of the ACL-RSI.
- Step 2: A bilingual reviewer compared the two translations for consistency with the original English version, identifying and resolving any conceptual discrepancies to create an initial Thai translation.
- Step 3: Two native English speakers proficient in Thai, who were unaware of the study’s purpose and had no access to the original English questionnaire, independently performed a back-translation.
- Step 4: A multidisciplinary committee consisting of an orthopedic surgeon, a methodologist, a sports physiotherapist, and a language expert compared the back-translated version with the original English questionnaire, finalizing the Thai ACL-RSI scale (Figure 1).
- Step 5: A pilot test was conducted with 10 eligible patients to assess clarity and comprehension, refining the questionnaire if necessary.

Phase 2 focused on validation and reliability testing, involving 40 Thai patients aged 18–50 years who had undergone unilateral ACLR. Eligibility criteria included primary ACLR using either hamstring tendon autograft (HTG) or patellar tendon autograft (PTG) performed between 6 months and 3 years prior, adherence to post-operative rehabilitation, clearance for return to play, participation in individual sports at least weekly, a pre-injury Tegner activity score of ≥5, and literacy in Thai. Exclusion criteria included bilateral ACLR, ACLR revision, multi-ligament reconstruction, or concurrent meniscus or cartilage repair. The ACL-RSI subscales consist of emotion, performance confidence, and risk assessment, which were each evaluated through 12 questions scored on a visual analog scale (VAS) ranging from 0 to 100 in 10-point increments. The total score was calculated by dividing the cumulative score of all 12 questions by a percentage, with higher scores reflecting a more favorable psychological response [5]. Additionally, the Thai version of the Tampa Scale of Kinesiophobia (Thai-TSK) was used as a reference questionnaire to gauge the fear of movement or injury among individuals experiencing chronic pain. The content validity was statistically assessed through the item-objective congruence index (IOC), internal consistency was evaluated using Cronbach’s alpha, test-retest reliability was measured via the intraclass correlation coefficient (ICC), and construct validity was scrutinized through Pearson correlation coefficients (*r*).

All statistical analyses were conducted using SPSS Statistics version 28.0.0, with participant characteristics presented descriptively through means, standard deviations, and frequencies. A content validity index (IOC) exceeding 0.5 indicated robust validity [6], while Cronbach’s alpha values between 0.70 and 0.95 signified satisfactory internal consistency [4, 14]. Test-retest reliability was characterized by ICC values, categorized as excellent (*r* > 0.81), very good (0.61 ≤ *r* ≤ 0.80), good (0.41 ≤ *r* ≤ 0.60), fair (0.21 ≤ *r* ≤ 0.40), and poor (0.00 ≤ *r* ≤ 0.20) [6]. Construct validity was ascertained via Pearson correlation coefficients (*r*), categorized as “strong” (*r* > 0.5), “moderate” (0.3 ≤ *r* ≤ 0.5), or “weak” (*r* < 0.3) to gauge the relationship between the Thai ACL-RSI and the Thai-TSK [6, 13].

## Results

Phase 1 did not reveal any significant language issues in the Thai translation or the subsequent English back-translation. After initial testing, no modifications were deemed necessary, and all participants reported that the questions were easily comprehensible.

Phase 2 involved a final study cohort of 40 subjects (8 females, 32 males), with an average age of 30.2 ± 7.32 years, body weight of 70.7 ± 13.36 kg, height of 170.1 ± 6.53 cm, and a body mass index of 24.5 ± 3.74 kg/m^2^. The interval between surgery and evaluation averaged 8.43 ± 1.83 months. Among the participants, half were students, police officers, or soldiers, constituting 50% of the sample. The remaining participants were comprised of company owners, private citizens, and government officers, accounting for 17.5% of the total. Notably, 50% of the patients were regular football players, while athletics and basketball each constituted 17% of the participant pool. On average, participants had 9.3 years of sports experience, predominantly as amateur athletes. A quarter of them had participated in national events, and 12.5% had competed in international tournaments (Table 1).

**Table 1:** Demographic data.

Patient data (*n* = 40)	Number	Percent
Gender		
Male	32	80
Female	8	20
Period from surgery to evaluation (year)	8.4 ± 1.83	
Occupation		
Student	10	25
Private officer	7	17.5
Government officer	7	17.5
Business owner	7	17.5
Policeman/Soldier	9	22.5
Type of sports		
Football	20	50
Athletic	5	12.5
Basketball	5	12.5
Cycling	3	7.5
Swimming	2	5
Badminton	2	5
Others	3	7.5
Duration of sport experience (year)	9.2 ± 8.25	
Highest level of sports participation		
International	5	12.5
National	6	15
Local	2	5
Not at all	27	67.5

Content validity was assessed using the index of IOC by a panel of three experts (one surgeon and two research methodologists). The IOC score was determined to be 0.91, signifying high content validity. The translated scale exhibited “excellent” internal consistency, with a Cronbach’s alpha coefficient of 0.84, indicating strong interrelatedness among the 12 items. The initial Thai ACL-RSI score was 63.39 points, and the retest yielded a score of 63.22 points, demonstrating great reliability. The test-retest analysis yielded an ICC of 0.75 (95% CI 0.58–0.86), indicating a strong degree of reliability.

The overall score of the Thai ACL-RSI displayed a negative correlation with the Thai-TSK (*r* = −0.671, *p* < 0.005). In individual dimensions, the Thai ACL-RSI demonstrated a significant negative correlation with the emotion and risk appraisal domains of the Thai-TSK (*p* < 0.001) (Table 2).

**Table 2:** The Pearson’s correlation between the Thai ACL-RSI and the Thai Tampa Scale of Kinesiophobia (Thai-TSK).

ACL-RSI domains	Score (Mean ± SD)		Pearson’s correlation	*p*-value
	Thai ACL-RSI	Thai-TSK		
Emotion	35.01 ± 8.1	11.51 ± 3.08	−0.605	<0.001*
Confidence	28.90 ± 10.1	15.52 ± 3.82	−0.108	0.339
Risk appraisal	11.93 ± 3.1	1.9 ± 0.8	−0.428	<0.001*
Total	63.45 ± 9.3	33.07 ± 8.85	−0.671	<0.001*

* Significant *p*-value < 0.005.

## Discussion

The main objective of this study was to conduct a cross-cultural translation of the ACL-RSI scale, namely from English to Thai. The primary finding of our study was the Thai version of the ACL-RSI scale had a high degree of internal consistency and reliability, comparable to the original form when administered to patients who had undergone ACLR. This study’s rigorous cross-cultural translation methodology guarantees the accuracy of meaning transfer from the English source text to the Thai target text. Furthermore, our research has established strong content validity by obtaining consensus among three experts, as evaluated by the index of IOC.

Our analysis revealed excellent internal consistency within the Thai ACL-RSI, exemplified by Cronbach’s alpha coefficient of 0.84. This degree of consistency aligns favorably with prior translations of the ACI-RSI, such as the Turkish (0.88) [9], Swedish (0.95) [7], French (0.96) [8], and Dutch (0.94) [10] versions. Furthermore, the test-retest reliability demonstrated marked strength, as indicated by an ICC of 0.75, which, while strong, falls slightly below the threshold for “excellent” reliability (ICC > 0.9) [15]. This places our findings in the same category as the Swedish ACL-RSI study (ICC = 0.89) [7] while being slightly lower than the Turkish (0.92) [9], French (0.99) [8], and Dutch (0.93) [10] translations. This moderate ICC value (0.75) might be attributed to the disparity among different ACL-RSI domains, as observed in our correlation analysis (Table 2). Specifically, while emotions and risk appraisal showed strong and moderate significant correlations with the Thai-TSK, confidence displayed a weak and statistically non-significant correlation. These variations suggest that the self-confidence domain might be influenced by additional factors not captured by the Thai-TSK, such as external social and environmental influences. Future refinements of the Thai ACL-RSI could explore alternative measures to assess self-confidence more comprehensively.

Previously validated for knee osteoarthritis patients following cross-cultural adaptation [13], the simplified Thai Tampa Scale of Kinesiophobia (Thai-TSK) provided a pertinent tool for assessing kinesiophobia (fear of movement) in ACLR patients. This psychological aspect assumes significance as it can influence post-operative individual sports capacity. TSK scores have been associated with a reduced likelihood of returning to physical activities after ACL rupture [16]. Consequently, the Thai version of the TSK established the concurrent validity of the Thai ACL-RSI. This study’s findings showed a negative correlation between the Thai ACL-RSI and Thai-TSK (*r* = −0.671, *p* < 0.001), akin to previous literature [7, 9, 10]. The correlation was most pronounced in the Swedish translation of ACL-RSI (*r* = −0.689, *p* < 0.001). This discrepancy might be attributed to the intrinsic psychological nature of the Thai-TSK, encompassing self-awareness and self-confidence. The study pinpointed a significantly negative correlation in the emotional component of the risk appraisal domain, affirming the pivotal role of psychological factors in resuming sports activities.

While previous studies had reported the influence of ACLR on physical knee function [17], psychological factors that impede an athlete’s return to play have been accorded relatively less attention. This gap is significant since a considerable proportion of athletes remain unable to fully regain their pre-injury performance level even after successful rehabilitation [18, 19]. Decisions regarding return to play remain intricate, with no established consensus in this domain [20]. Recent research indicates that patients often lack psychological readiness to resume their previous performance level [21], primarily due to concerns of re-injury. Existing knee scoring systems for ACLR patients emphasize clinical function (e.g., KOOS and IKDC scores) and activity status (Tegner score) but overlook psychological aspects.

Additionally, to further contextualize our findings, comparisons with other ACL-RSI versions could be valuable. Studies have validated ACL-RSI translations in various languages, including Italian [22], Brazilian Portuguese [23], Japanese [24], and Lithuanian [25]. Incorporating these versions in future meta-analyses could help delineate cultural influences on psychological readiness post-ACLR.

For the clinical implication, the Thai ACL-RSI scale can assist Thai sports medicine practitioners in assessing patients’ readiness to return to physical activity, especially those grappling with psychological impediments. Thus, a comprehensive evaluation of psychological aspects becomes indispensable, warranting psychological intervention alongside physical assessment during the postoperative rehabilitation phase.

This study has some limitations. First, the small sample size (40 participants) may limit generalizability. Future research should include larger cohorts for better statistical power. Second, ACL-RSI scores may not change significantly beyond 12 months post-ACLR [15], affecting internal validity. Future studies should assess score trends over time. Third, using only the Thai TSK limits psychological assessment. Additional tools like the Injury-Psychological Readiness to Return to Sport Questionnaire or IKDC Subjective Knee Form could improve validation. Fourth, while the Thai TSK is validated in ACL studies, it was designed for chronic pain, so alternative psychological measures should be considered. Finally, the male-dominated sample may limit applicability to female athletes. Future studies should explore gender differences and validate the Thai ACL-RSI for other knee injuries.

## Conclusion

The Thai ACL-RSI scale exhibited great validity, reliability, and consistency among individuals who had undergone ACL reconstruction. The utility of this instrument is gauging the impact of psychological variables on sports activities post-ACLR that may affect the clinical treatment outcomes and be useful in individual rehabilitation for each patient.

## Data Availability

The data that support the findings of this study are available from the corresponding author upon reasonable request.
